# Methylation status of TSHr in well-differentiated thyroid cancer by using cytologic material

**DOI:** 10.1186/s12885-015-1861-1

**Published:** 2015-10-30

**Authors:** Kinyas Kartal, Sevgen Onder, Kemal Kosemehmetoglu, Sadettin Kilickap, Yesim Gaye Tezel, Volkan Kaynaroglu

**Affiliations:** 1 Department of General Surgery, Sisli Hamidiye Etfal Training and Research Hospital, Istanbul, Turkey; 2 Department of Pathology, Hacettepe School of Medicine, Ankara, Turkey; 3 Department of Preventive Oncology, Hacettepe School of Medicine, Ankara, Turkey; 4 Department of General Surgery, Hacettepe School of Medicine, Ankara, Turkey

## Abstract

**Background:**

The role of methylation status of the thyroid stimulating hormone receptor gene (TSHr) in the discrimination of benign and malignant thyroid nodules has already been studied using paraffin blocks and cell lines. As cytological sampling plays an important role in assessment of thyroidal nodules, we have investigated the potential clinical use of TSHr methylation status of fine needle aspiration specimens reported according to Bethesda System.

**Method:**

Sixty nine patients who had both cytological and pathological diagnosis of the same nodule were selected. Four groups were composed according to cytological and pathological diagnoses: Benign (B), papillary thyroid carcinoma (PTC), atypia of unknown significance (AUS) and follicular neoplasia (FN). The latter 2 groups were further sub-classified into 2 as benign (AUS-B and FN-B) and malignant (AUS-M and FN-M) according to final pathological diagnosis. DNAs were isolated from the fine needle aspiration cytology specimens and the methylation status of TSHr promotor region was investigated by using methylation specific polymerase chain reaction.

**Results:**

Overall, TSHr methylation was present in 58 % of cases; 71 % of malignant and 46 % of benign nodules. PTC group showed the highest TSHr methylation rate (87 %), followed by 61 % in AUS, 44 % in B, and 30 % in FN (*p* = 0.016). TSHr methylation rate was significantly higher in PTC group when compared to B (*p* = 0.013) and FN-B (*p* = 0.004) groups; but not in FN-M (*p* = 0.115) or AUS (*p* = 0.096) groups. All 9 cases of papillary thyroid carcinoma with lymph node metastasis showed TSHr methylation. Positive predictive value, negative predictive value, sensitivity and specificity of TSHr methylation in determination of malignancy were calculated as 60, 66, 71 and 54 %, respectively.

**Conclusion:**

The eminent ratio of TSHr methylation in well-differentiated thyroid carcinoma against benign thyroidal nodules adduced that TSHr methylation status can be utilized as a tumor marker for well-differentiated thyroid cancer; however, it has a limited value. The determination of methylation status of TSHr gene had no efficiency on decision of the malignant potential for the nodules which are cytologically classified as atypia of undetermined significance.

## Background

Thyroid cancer is the most common endocrine malignancy, comprising 90 % of endocrine cancer, although it represents less than 1 % of all human cancer. The most prevalent types of thyroid cancer are well differentiated (papillary or follicular) thyroid carcinomas [1]. Introduction of fine needle aspiration cytology (FNAC) has dramatically changed the surgical management of the patients, and FNAC has become the most reliable test for the diagnosis of the thyroid cancer. In order to establish a good correlation between surgeon and pathologists, the cytological information obtained from FNAC is assembled into the 6 groups according to the Bethesda System: 1) Non-diagnostic, 2) Benign, 3) Atypia of Undetermined Significance or Follicular Lesion of Undetermined Significance, 4) Follicular Neoplasm or Suspicious for a Follicular Neoplasm, 5) Suspicious for Malignancy and 6) Malignant [2]. Consequently, the management of patients with FNAC resulted as “suspicious for malignancy” or “follicular lesion” has arisen as a contemporary issue in the treatment of thyroid cancer. Therefore, scientific efforts are directed to increase diagnostic precision in the “atypia of undetermined significance”and “follicular neoplasia” groups in order to achieve a more reliable surgical management.

It is known that methylation of specific genes, which silence the affected gene, plays an important role in the pathogenesis of thyroid cancer as in any other type of cancers [3]. As thyroid stimulating hormone receptor is the main hormone receptor responsible for growth and physiological functioning of thyroid gland, methylation of TSHr gene is believed to have an important role in the development of thyroid cancer [4, 5]. In fact, there are some studies about histological material of thyroid nodules revealing the role of TSHr methylation in the thyroid carcinogenesis [6–9]; however, data about the TSHr methylation status in FNAC material is lacking.

In this study, we investigated the methylation status of TSHr in well differentiated thyroid cancers using cytology derived DNA, hence its potential role as a diagnostic test in the discrimination of benign and malignant thyroid nodules.

## Methods

This study was approved by the ethics committee of Hacettepe University. Study participants or their legal guardians provided written informed consent.

### Patient groups and cytological material

Eighty-eight patients, who had cytological diagnosis, surgical intervention and final pathological evaluation in our center, were selected from 4355 consecutive patients, who presented with thyroidal nodule between 2007 and 2011. Vast majority of the FNAC in our institution was performed under ultrasound guidance. FNAC material was obtained from the archives; 19 patients were excluded because of limited diagnostic cytological material. Slides of remaining 69 patients were reviewed by two pathologists (SO and KK). Cytological diagnoses were given by a single pathologist (SO). Clinical features, thyroglobulin levels, nodule size and the results of cytology and pathology reports were recorded using patient files and computer-based patient data system. Only cytological slides were used for the study, and 1 to 3 slides for each case were selected regarding to the availability of the cytological specimen and cellularity.

According to cytology and pathology reports, 69 patients were classified into 4 groups: 1) Group “B”, patients with benign FNAC treated surgically because of a thyroid nodule greater than 3 cm, patient’s decision or cosmetic purposes, and diagnosed as benign pathologically, 2) Group “PTC”, patients with malignant FNAC and pathologically diagnosed as well-differentiated papillary thyroid carcinoma, 3) Group “AUS”, patients diagnosed as atypia of undetermined significance in FNAC, 4) Group “FN”, patients evaluated as follicular neoplasm in FNAC. The 3rd and 4th groups were further divided into 2 subgroups as benign (AUS-B and FN-B) and malignant (AUS-M and FN-M) according to final pathological assessment. Patient selection was supervised in order to have an equal number in each group (Table 1).

**Table 1:** Patient groups

Groups	FNAC	Pathological Diagnosis	*n*
B	Benign	Benign	16
PTC	Papillary thyroid carcinoma	Papillary thyroid carcinoma	15
AUS			
AUS-B	AUS	Benign	15
AUS-M	AUS	Papillary thyroid carcinoma	13
FN			
FN-B	Suspicious for Follicular neoplasia	Benign (follicular adenoma)	4
FN-M	Suspicious for Follicular neoplasia	Malignant (follicular carcinoma)	6

*FNAC* Fine needle aspiration cytology, *n* number of patients, *AUS* atypia of undetermined significance, *FN* Follicular neoplasm, *AUS-B* atypia of undetermined significance – benign, *AUS-M* atypia of undetermined significance – Malignant, *FN-B* Follicular adenoma, *FN-M* Follicular carcinoma

### TSHr methylation by methylation specific polymerase chain reaction (MSP)

One hundred forty slides from 69 patients were selected for DNA isolation. DNAs were isolated and bi-sulfated using CpGenoma DNA Modification Kit S7820 (Chemicon International Company, USA) according to the manufacturer’s protocols. The sequence of the primers used in the DNA amplification were obtained from the study of Smith et al. [6]. 2.25 ng from each of 3 % Dimethyl Sulfoxide (DMSO), 16.6 mM Ammonium Sulfate, 67 mM Tris- Borate- EDTA buffer (Santa Cruz, USA, pH 8.8), 6.7 mM MgCl_2_, 10 mM 2-Mercaptoethanol, 1.23 mM deoxynucleotide triphosphates, sense and anti-sense DNA base primers, 1 μl from bi-sulfite modified DNA and 0.5 units from DNA Taq polymerase (Invitrogen Comp., USA) were mixed for MSP.

**Fig. 1:**
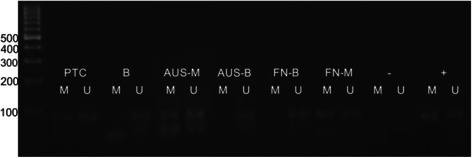
Elecrophoresis gel appearance of TSHr methylation status

The PCR conditions for the TSHr were as follows: 1 cycle at 95 °C for 15 min, 95 °C for 45 s, 61 °C for 45 s and 72 °C for 1 min. This cycle was repeated for 40 times followed by a final 10 min extension at 72 °C. Positive and negative control DNA samples and controls without DNA were used for each set of reactions. The final PCR products were resolved with agarose gel electrophoresis for 1 h and stained with ethidium bromide for visualization (Major Science, DI-01 model). Bands at 91 and 88 base pairs were regarded as references for unmethylated and methylated TSHr, respectively (Fig. 1) [7].

### Statistics

Patients’ variables were analyzed using SPSS 21.0. The relationships in between categorical variables were analyzed by either Pearson chi-square or Fisher’s exact test. Since the data did not meet the assumption of normal distribution (by Kolmogorov-Smirnov test), the information between two independent groups was analyzed with the Mann–Whitney *U*-test or Kruskal-Wallis test. All statistical inferences were two-tailed and *p* value <0.05 was considered to be statistically significant.

## Results

Clinical information is summarized in Table 2. Fifty-nine patients were female (85.5 %) and 10 were male (14.5 %). Male to female ratio of B and AUS groups were lower than that of PTC and FN groups, particularly of FN-B group. The mean age was49 (range 26–80), and the age was evenly distributed among groups. Mean diameter of nodules was 23.0 ± 11.6 mm; B and FN groups had larger nodules than PTC and AUS groups (*p* = 0,001). Nine patients in PTC group had at least 1 metastatic lymph node. Thyroglobulin levels were not significantly different among the groups (*P* = 0.326).

**Table 2:** Clinical data

Group	Age (mean ± SD)	Sex (M:F)	Ultrasonographic diameter of nodule (mean ± SD, mm)	Serum Thyroglobulin [median, mg/dl (IQR)]	Cases with high (>50 mg/dl) thyroglobulin (%)
B	50 ± 14	1:15	26 ± 9	47.8 (20.0–160.3)	50
PTC	40 ± 10	4:11	14 ± 8	9.3 (0.2–98.0)	33
AUS	52 ± 13	2:26	22 ± 12	33.0 (1.3–119.0)	36
AUS-B	55 ± 13	1:14	22 ± 13	41 (0.3–135.5)	39
AUS-M	48 ± 13	1:12	23 ± 11	20 (3.1–96.5)	33
FN	54 ± 14	3:7	33 ± 11	16.7 (3.0–66.3)	25
FN-B	50 ± 14	2:2	28 ± 5	8.4 (2.6–32.7)	0
FN-M	57 ± 15	1:5	36 ± 13	35.4 (5.7–159.0)	50

*M* Male, *F* Female, *IQR* Interquartile range, *SD* Standard deviation

Overall TSHr methylation was positive in 58 % of cases; 71 % of malignant nodules and 46 % of benign nodules showed TSHr methylation (Fig. 1, *p* = 0.036). Positive predictive value, negative predictive value, sensitivity and specificity of TSHr methylation In FNAC material in determination of malignancy were calculated as 60, 66, 71 and 54 %, respectively. There was no correlation between TSHr methylation status and serum thyroglobulin levels (*p* = 0.151), age (*p* = 0.162) or size of the nodule (*p* = 0.892).

**Fig. 2:**
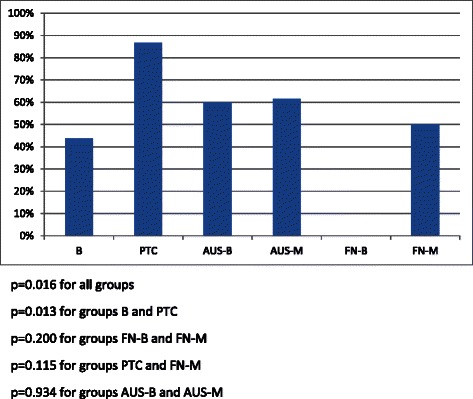
TSHr methylation status of diagnostic groups

The highest rate of TSHr methylation was found in the PTC group (13/15, 87 %), it was 44 % (7/16) in B, 61 % (17/28) in AUS and 30 % (3/10) in FN (*p* = 0.016, Fig. 2). There were no cases of follicular adenomas with a positive methylation status of TSHr(0/4) whereas in 3 of 6 (50 %) follicular carcinomas TSHr’s were found to be methylated. Papillary thyroid carcinomas (PTC group) significantly showed higher TSHr methylation status than benign colloidal nodules (B group, *p* = 0.013) and follicular adenomas (FN-B, *p* = 0.004) do; but no higher than in follicular carcinomas (FN-M, *p* = 0.115) or in AUS (*p* = 0.096). In PTC group, all 9 cases showing lymph node metastasis had positive methylation status of TSHr. There was no significant difference between subgroups of atypia of unknown significance (*p* = 0.934). TSHr of follicular carcinomas tended to be methylated much more frequently than of follicular adenomas, but it did not reach the level of significance (50 % vs 0 %; *p* = 0.200).

## Discussion

The association between well-differentiated thyroid carcinomas and TSHr methylation status was previously studied using formaldehyde-fixed paraffin-embedded thyroid tissues or thyroid cell lines [3, 6–9]. However, its potential diagnostic impact on thyroid FNAC was not investigated elsewhere. In our study, we found that a significant number (over half) of patients with thyroid nodule showed TSHr methylation using cytological material. Unsurprisingly, papillary thyroid carcinomas tended to show more frequent TSHr methylation compared to benign thyroidal nodules or follicular adenomas. Moreover, we demonstrated a higher frequency of TSHr methylation in follicular carcinomas than in follicular adenomas, although the difference did not reach statistical significance possibly due to low number of studied cases. Regarding the fact that thyroid FNAC and Bethesda classification have a major role in the surgical management of thyroid nodules, the assessment of TSHr methylation status may be a useful marker adjunct to the cytological examination, in particular, aiding to differentiate papillary thyroid carcinomas from colloidal nodules, as well as follicular adenomas from follicular carcinomas. The results of our study also indicate that the evaluation of TSHr methylation status has no impact on the cases diagnosed as “atypia of undetermined significance” of Bethesda classification. Because the sensitivity and specificity of TSHr methylation for detecting malignancy is found to be around 60–65 %, its direct use as a marker of malignancy is not recommended.

According to study of Smith et al., in which gene-specific methylation status of TSHr, ECAD, NIS- L, ATM and DAPC receptors were studied, methylation rates for NIS-L, ATM, ECAD, and TSHr were higher in papillary thyroid carcinoma compared to the control group, including colloidal nodules, follicular adenoma, and non-neoplastic thyroid tissue [6]. TSHr methylation rate for papillary thyroid carcinoma was only 34 % (11/32), approximately one third of our rate in PTC group reaching 90 % in our study. Intriguingly, they also observed TSHr methylation in 20 % (2/10) of follicular adenomas. The results of Xing et al. [7] are more comparable with our findings: none of normal thyroid tissue or adenomas had TSHr in the methylated form, whereas 59 % of papillary thyroid carcinomas and 47 % of follicular carcinomas showed a TSHr methylation. Similarly, Dai et al. demonstrated that 68 % of papillary thyroid carcinomas harbored TSHr methylation among Chinese population [8]. These differences in the methylation status of TSHr between our study and the others can be explained on several grounds. Firstly, cytopathological preparations we used for the DNA isolation was superior to the histological material that is formalin-fixed paraffin-embedded tissue used by the previous studies. Second, regional differences affecting both genetic and environmental properties may also account on deviation of the TSHr methylation status. Field effect may have an impact on the microenvironment of thyroid tissue and the development of epigenetic changes, regarding to the finding that TSHr methylation status of the neoplastic nodule with surrounding non-neoplastic thyroid tissue was comparable [8]. Lastly, methodology can lead to slight diversities.

Based on the high rate of TSHr methylation in papillary thyroid carcinoma, some authors investigated the effect of de-methylating agents in the treatment of thyroid malignancies. Kim et al. demonstrated 90 % regression of oncogenetic differentiation and delay in the thyroid tumor growth in the group treated with *5′-aza-2′deoxynucleotide*, a de-methylating agent [9]. Regarding to the fact that all papillary thyroid carcinomas with metastasis have shown to bear TSHr methylation in our study, patients with metastatic disease may become the first candidates for such a potential therapy with de-methylating agents in the future. Smith et al. found that tumors recurred less in patients with methylated TSHr; however, TSHr methylation status in papillary thyroid carcinoma needs to be further investigated for its prognostic value.

## Conclusion

According to our study, TSHr methylation status could be used as a diagnostic test in patients with well differentiated thyroid carcinoma especially in “suspicious for follicular neoplasia” and “papillary thyroid carcinoma” groups; however, low sensitivity and specificity for determination of carcinoma, limits its diagnostic usefulness. TSHr in the methylated form is always present in metastatic papillary thyroid carcinomas, therefore TSHr methylation status may be used as a prognostic factor and as a potential predictive factor for the treatment modalities such as de-methylating agents in the future.

## Abbreviations

TSHr: Thyroid stimulating hormone receptor
PTC: Papillary thyroid carcinoma
AUS: Atypia of unknown significance
FN: Follicular neoplasia
FNAC: Fine needle aspiration cytology
